# Determinants of Operative Time in Arthroscopic Rotator Cuff Repair

**DOI:** 10.3390/jcm12051886

**Published:** 2023-02-27

**Authors:** Daniel J. Stitz, Allen A. Guo, Patrick H. Lam, George A. C. Murrell

**Affiliations:** 1 Orthopaedic Research Institute, St. George Hospital Campus, Kogarah, NSW 2217, Australia; 2 School of Medicine, University of New South Wales, Sydney, NSW 2052, Australia

**Keywords:** rotator cuff repair, operative efficiency, surgeon experience, undersurface technique, operative duration, surgical throughput

## Abstract

Arthroscopic rotator cuff repairs have been reported to take between 72 and 113 min to complete. This team has adopted its practice to reduce rotator cuff repair times. We aimed to determine (1) what factors reduced operative time, and (2) whether arthroscopic rotator cuff repairs could be performed in under 5 min. Consecutive rotator cuff repairs were filmed with the intent of capturing a <5-min repair. A retrospective analysis of prospectively collected data of 2232 patients who underwent primary arthroscopic rotator cuff repair by a single surgeon was performed using Spearman’s correlations and multiple linear regression. Cohen’s f^2^ values were calculated to quantify effect size. Video footage of a 4-min arthroscopic repair was captured on the 4th case. Backwards stepwise multivariate linear regression found that an undersurface repair technique (f^2^ = 0.08, *p* < 0.001), fewer surgical anchors (f^2^ = 0.06, *p* < 0.001), more recent case number (f^2^ = 0.01, *p* < 0.001), smaller tear size (f^2^ = 0.01, *p* < 0.001), increased assistant case number (f^2^ = 0.01, *p* < 0.001), female sex (f^2^ = 0.004, *p* < 0.001), higher repair quality ranking (f^2^ = 0.006, *p* < 0.001) and private hospital (f^2^ = 0.005, *p* < 0.001) were independently associated with a faster operative time. Use of the undersurface repair technique, reduced anchor number, smaller tear size, increased surgeon and assistant surgeon case number, performing repairs in a private hospital and female sex independently lowered operative time. A <5-min repair was captured.

## 1. Introduction

### 1.1. Background

Arthroscopic rotator cuff repair was the longest upper limb day procedure in the USA in 2006 [1]. Lower hospital volume, surgeon inexperience, male sex and increased body mass have been reported to increase operative duration in rotator cuff repairs [2,3,4,5,6]. Each extra minute of operative time has been estimated to add a further 26 USD to the cost of the procedure [7]. Prolonged operative time has also been associated with a higher rate of surgical complications, readmission and retear in rotator cuff repairs [8,9,10,11]. As such, reducing operative time is associated with significant financial and clinical benefit.

A number of predominantly US-based groups have reported average operative times for arthroscopic rotator cuff repairs ranging from 72 to 113 min [3,4,7,12,13,14,15,16,17,18,19]. Our group by comparison has reported arthroscopic rotator cuff repairs being performed on average in 16–20 min and as fast as 4 min. This 4-min figure and the discrepancy between our and other groups’ operative time has often generated scrutiny from reviewers when assessing our publications during the peer-reviewed submission process.

### 1.2. Aims

The aim of this study, therefore, was two-fold:

(1) To determine what factors were associated with faster rotator cuff repairs in our practice.
(2) To determine whether arthroscopic rotator cuff repairs can be performed in under 5 min.

## 2. Materials and Methods

### 2.1. Aim 1: Determinants of Operative Time

A retrospective cohort study was performed using prospectively collected operative report and clinical history data to post hoc analyse which factors impacted operative time.

#### 2.1.1. Inclusion and Exclusion Criteria

Data from patients were included if they underwent a primary arthroscopic rotator cuff repair by the surgeon performed either in isolation or in conjunction with acromioplasty, biceps tenodesis, manipulation under anaesthesia or capsular release. A primary repair was defined as a rotator cuff that had not undergone previous surgical treatment. Exclusion criteria included rotator cuff repair cases that involved a polytetrafluoroethylene patch, revision repairs or if the repair was performed concurrently with other surgical procedures (capsular release, stabilisation, debridement of calcific tendonitis or fracture reduction). Cases where the tendon was irreparable or only partially repaired, patients that failed to return for 6-month follow-up examination and those that were missing operative time data were also excluded. 

#### 2.1.2. Pre-Operative Data

Prior to undergoing surgery, patients completed standardised questionnaires wherein they nominated the onset of their symptoms and whether their rotator cuff tear was work-related and/or caused by a specific injury.

#### 2.1.3. Operative Procedure

**Figure 1:**
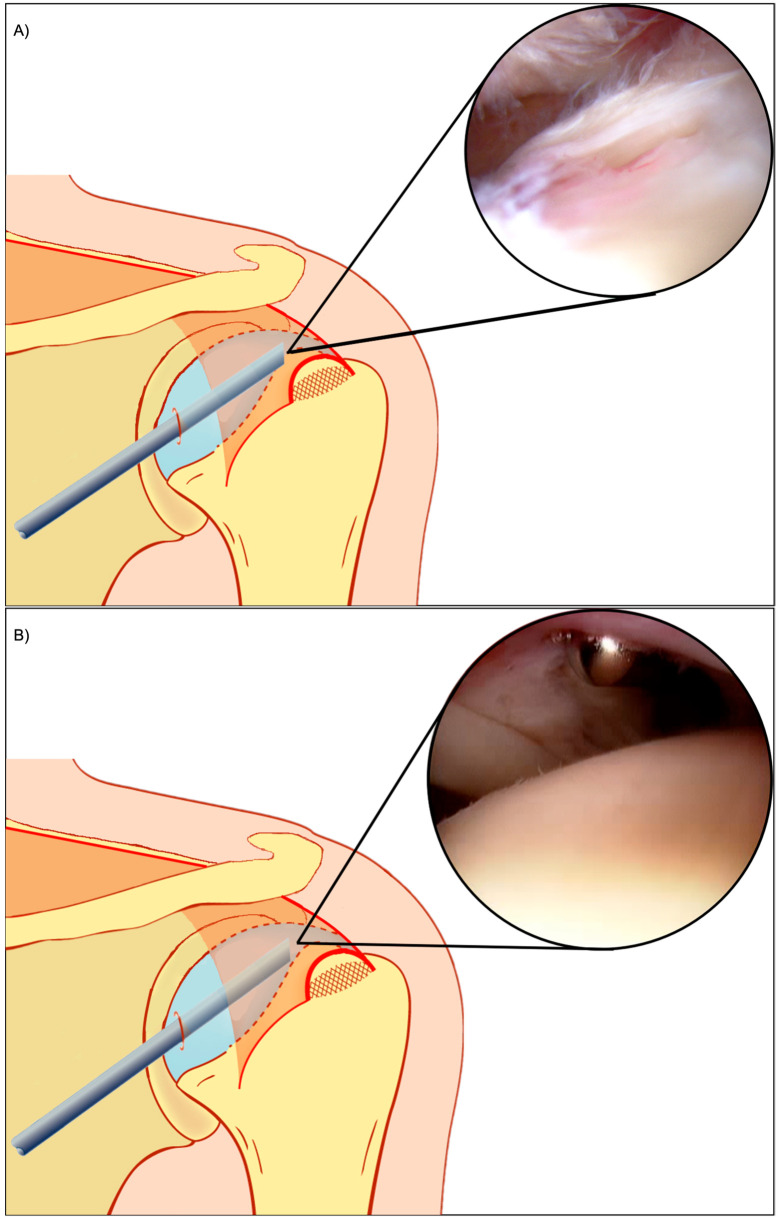
(**A**) Arthroscopic rotator cuff repair visualised from the bursal side. (**B**) Arthroscopic rotator cuff repair visualised from the undersurface. Adapted from Wu et al. [20].

Patients were administered an interscalene block and light sedation before being positioned in the beach chair position. Following prepping and draping, an arthroscope was inserted into a posterior portal, approximately 2 cm medial, 2 cm inferior from the posterolateral edge of the acromion. Undersurface repairs visualised the torn tendon from beneath the rotator cuff (Figure 1A), whilst bursal-sided repairs visualised the torn tendon from the subacromial bursa (Figure 1B).

A spinal needle was utilised to identify the location of a lateral portal, approximately 2 cm lateral from the midline of the lateral edge of the acromion. A portal was created at this site with an 11 blade to allow for entry of arthroscopic tools. Once the torn edge was visualised, a 4.0 or 5.5 mm arthroscopic shaver was inserted to debride the torn tendon edge as well as the greater tuberosity. An Opus Smart-Stitch^®^ Suture Device (Smith and Nephew, Watford, UK) was inserted through the lateral portal and used to deliver a #2 polyester inverted mattress suture through the torn tendon edge. A T-handled punch and mallet was used to create a hole for the repair anchor on the greater tuberosity. The ends of the sutures were used to load an Opus Magnum 2 Knotless Anchor Device (Smith & Nephew, Watford, UK), which was then inserted into the hole created before the anchor was deployed. One or more sutures and anchors were utilised according to tear size. This technique necessitates use of a knotless anchor, as repair of the tear occludes vision of outside the capsule. Further detail regarding this procedure is summarised by Wu et al. [20].

#### 2.1.4. Operative Time

Operative time was recorded for each surgery using a digital stopwatch. Operative time was defined as time elapsed from insertion of arthroscope into the glenohumeral joint to initiation of skin closure.

#### 2.1.5. Intra-Operative Data

Intra-operatively, tear size was measured by estimating the anteroposterior and mediolateral lengths of the tear, using the diameter of the 4.0 or 5.5 mm shaver for reference. Tear thickness was measured by comparing the thickness of the tear against areas of healthy tendon, namely the bare area. In addition, tissue quality, tendon mobility and repair quality were graded fair, good, very good or excellent as previously described [21]. The number of anchors used in the repair, repair technique utilised (undersurface, bursal or both) and whether other concurrent procedures (acromioplasty, biceps tenodesis, manipulation under anaesthesia or capsular release) were also recorded as previously described [21]. Post hoc, case number was calculated sequentially from each operative report that met the inclusion and exclusion criteria. Case number for assistants and surgeons was defined as the number of surgeries that met inclusion and exclusion criteria previously performed by the surgeon or assistant surgeon, inclusive of that repair. 

#### 2.1.6. Repair Integrity

A single on-site ultrasonographer assessed repair integrity. Ultrasound was used to assess repair integrity at 6 months follow-up using a General Electric Logiq E9, with a high-frequency (12 MHz) linear transducer (Boston, MA, USA) or a Siemens ACUSON S2000 ultrasound system (Erlangen, Germany) utilising previously described examination protocols [22].

#### 2.1.7. Statistical Analysis

Statistical analysis was performed using SPSS (IBM, New York, NY, USA). Univariate correlation using Spearman’s test were performed between pre-operative and intra-operative variables against operative time. Of the factors that correlated, a backwards multivariate linear regression was performed against operative time to assess which factors were independently associated. A multivariate linear regression was also performed on just variables known pre-operatively to generate a predictive model for operative time. Cohen’s f^2^ values were calculated for factors found independent by multivariate regression to measure effect size, where f^2^ ≥ 0.02, f^2^ ≥ 0.15, and f^2^ ≥ 0.35 corresponded to small, medium and large effect sizes, respectively [23].

### 2.2. Aim 2: Capturing a Less Than 5-Min Rotator Cuff Repair

A Sony FDR-Ax33 Handycam (Tokyo, Japan) was used to capture external video footage of sequential arthroscopic rotator cuff repairs, with the intent of capturing a 4-min procedure. Arthroscopic footage was captured using a Smith and Nephew arthroscope (Watford, UK). Apple iMovie (Palo Alto, CA, USA) was used to combine arthroscopic and unedited macroscopic footage as well as add a voice-over describing the video footage.

## 3. Results

### 3.1. Aim 1: Determinants of Operative Time

#### 3.1.1. Cohort

**Figure 2:**
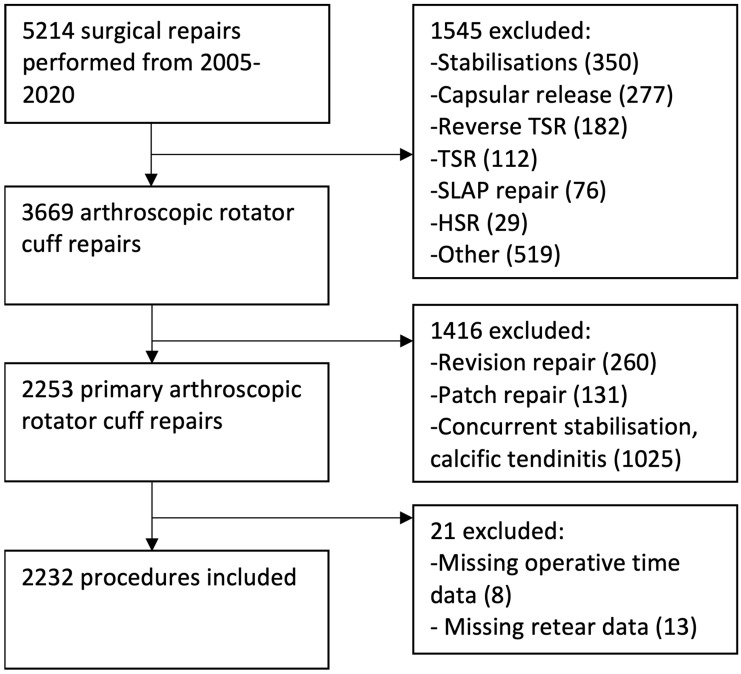
Inclusion flowchart. TSR = total shoulder replacement, SLAP = superior labral tear anterior to posterior, HSR = hemi-shoulder replacement.

Between 2005 and 2020, 5214 procedures were performed by the surgeon. These were patients referred to surgeon’s private and public practices. Of these 5214, 3669 were arthroscopic rotator cuff repairs. Of these, 260 were revision repairs; 131 were patch repairs and 1025 were performed in conjunction with arthroscopic stabilisation or calcific tendinitis repairs, resulting in 2253 repairs. Of these 2253, 21 were excluded as they were missing operative time or retear data, yielding 2232 repairs being included (Figure 2).

#### 3.1.2. Demographics

Of the 2232 patients who formed the study group, 1259 (56%) were male and 973 (44%) were female. The average (SD) age was 59 (11), ranging from 15 to 91. There were 1338 (60%) right shoulder cuff tears and 894 (40%) left shoulder tears. Tear size ranged from 4 to 6400 mm^2^, with 1724 (77%) of tears less than 500 mm^2^. Overall, 1711 patients were treated in private hospitals, 242 were treated in a public hospital, and 279 had operative reports which were not hospital specific. The average (SD) operative time was 21 (12) minutes, ranging from 3 to 97 min. In total, 1260 (56%) repairs were performed using the undersurface technique, 247 (11%) utilised a bursal-sided approach, 483 (22%) used both approaches. In addition, 242 (11%) did not list a repair technique on the operative report. At 6 months, 271 of 2232 (12%, 0–25%) tears had retorn (Table 1).

#### 3.1.3. Multivariate Analysis

Multivariate analysis was performed utilising factors found to be significant by univariate analysis using Spearman’s correlation with operative time as the dependent variable to determine which factors independently predicted operative time. The most important determinant of operative time was the use of the undersurface repair technique with a small–medium effect size (f^2^ = 0.08; *p* < 0.001) followed by a decreasing number of anchors used (f^2^ = 0.06; small-medium effect size, *p* < 0.001). Surgeon experience (case number), assistant experience (case number), female sex, higher ranked repair quality and smaller tear size were found to independently reduce operative time. This analysis generated a model for operative time with R^2^ = 0.4, *p* < 0.001 (Table 2).

#### 3.1.4. Effect of Surgical Technique

**Figure 3:**
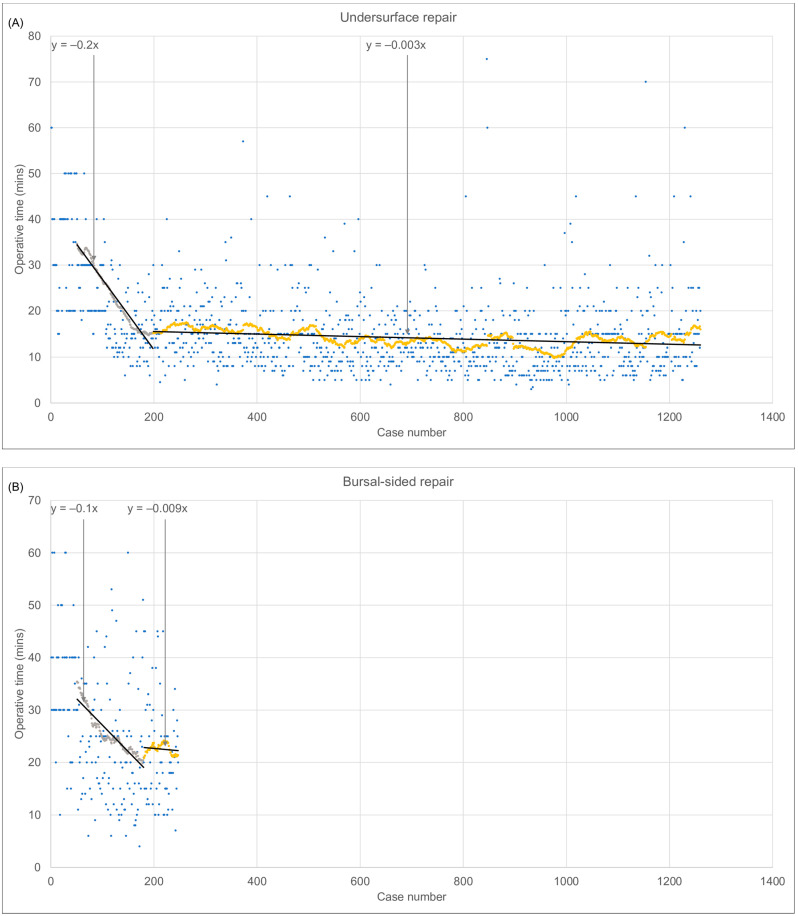

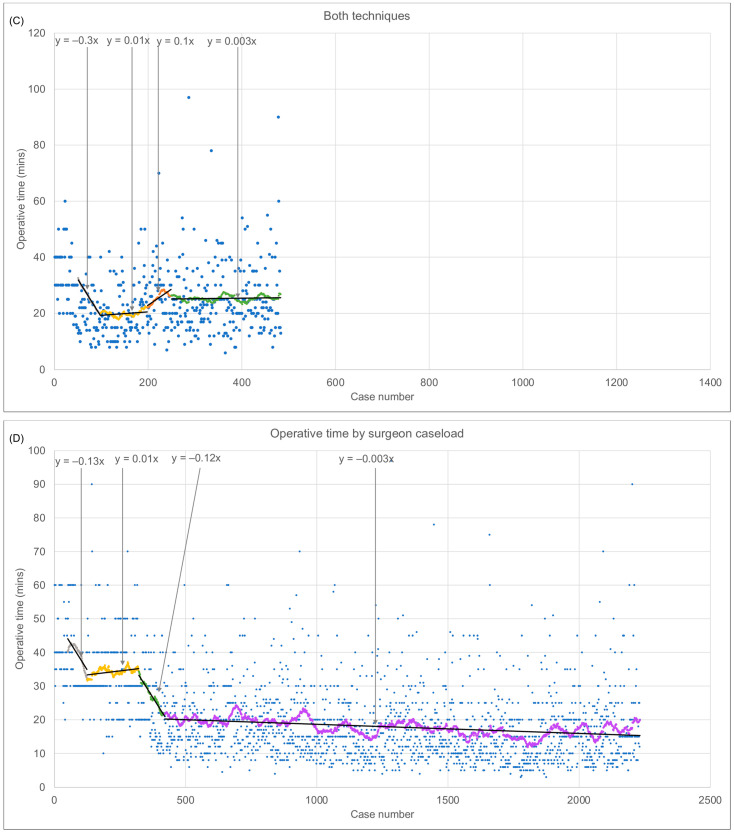

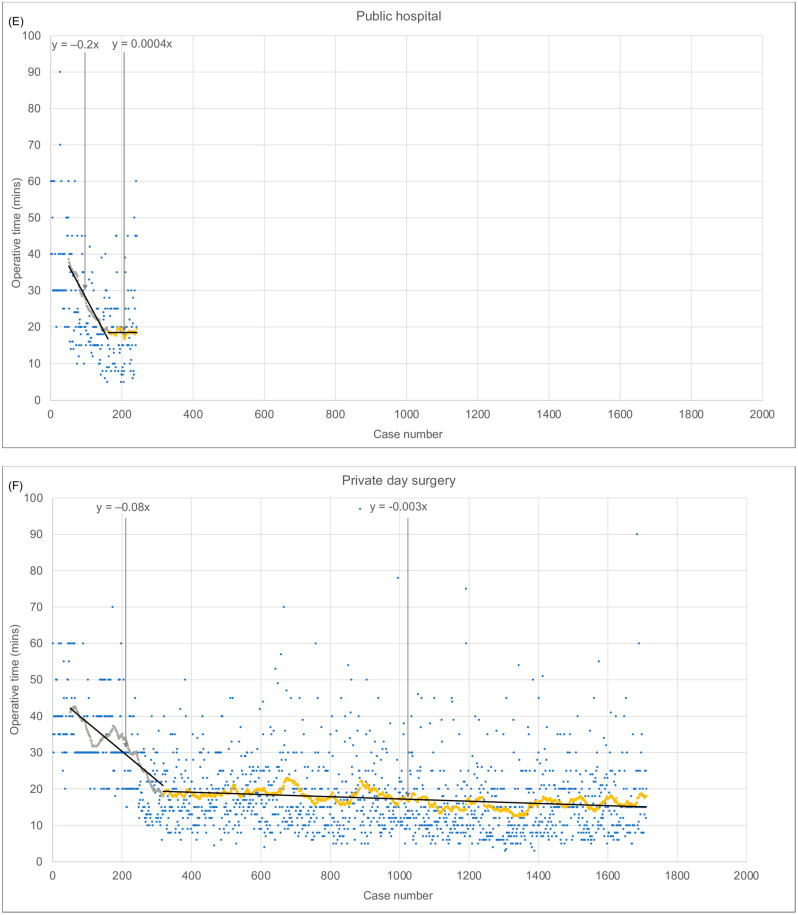
Rolling average operative time for (**A**) undersurface repairs, (**B**) bursal-sided repairs, (**C**) repairs utilising both techniques. Undersurface and bursal-sided repairs demonstrate a reduction in rolling average operative time between cases 0 and 200. (**D**) Rolling average operative time by surgeon caseload. Increased caseload showed a reduction in rolling average operative time. —Rolling average operative time for (**E**) public hospital cases and (**F**) private day surgery cases. Rolling average operative times reduced in both public and private hospitals, the private hospital was faster on average. Cases shown in blue, rolling average for 50 cases shown in grey, yellow orange, green and/or purple.

Undersurface repairs were faster and more time consistent compared to bursal-sided repairs and repairs utilising both techniques. The average (SD) operative time for undersurface repairs was 16 (9) minutes, whilst bursal-sided repairs took 25 (12) minutes, and both techniques took 25 (12) minutes. The rolling average operative time reduced the most during the first 200 cases of undersurface repairs (from 35 to 15 min) and bursal-sided repairs (from 35 min to 21 min). Following these initial 200 cases, reductions of less than 1 min were observed in the remainder of the undersurface (1260 cases) and bursal-sided (247 cases) repairs. In cases where both repair techniques were utilised, cases 0–100 were associated with a reduction (33 to 20 min), cases 100–250 were associated with an increase (22 to 26 min), and the remainder of the 483 cases were associated with a less steep increase (26 to 27 min) (Figure 3A–C).

#### 3.1.5. Number of Anchors

**Figure 4:**
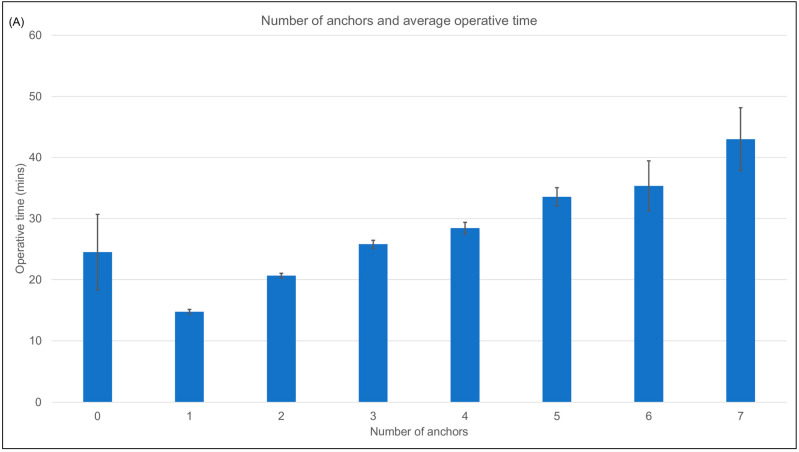

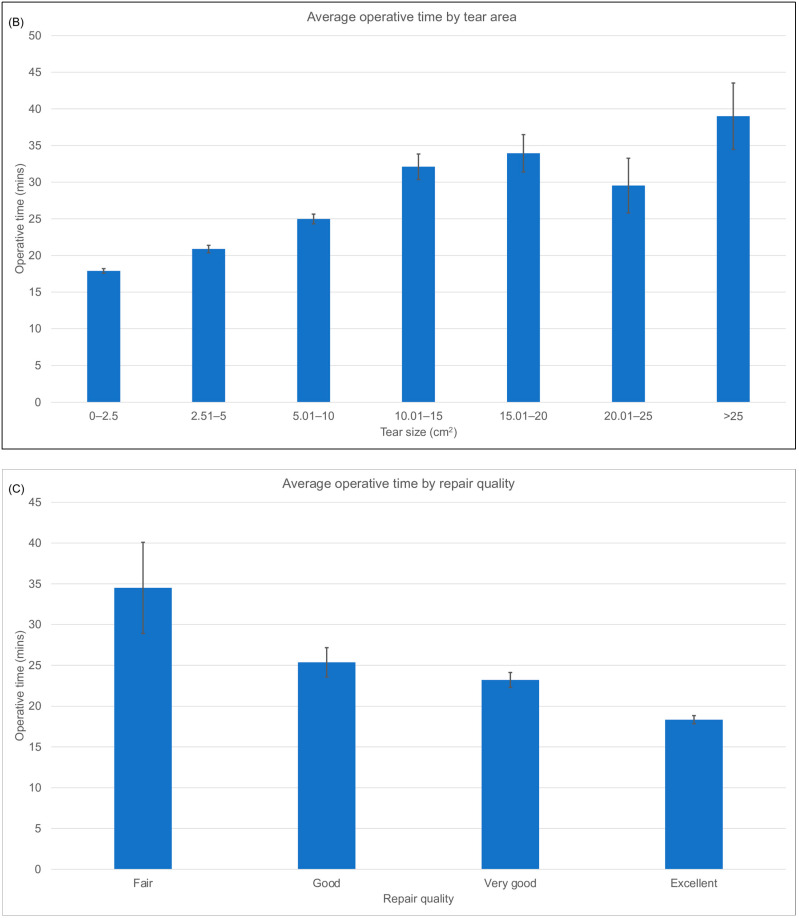
(**A**) Average operative time against number of anchors utilised in repair. Error bars indicate SEMs. Use of more anchors increased average operative time. (**B**) Average operative time against tear size. Error bars indicate SEMs. Increased tear size was associated with increased operative time. (**C**) Average operative time against repair quality. Error bars indicate SEMs. Higher repair quality was associated with reduced operative time.

Increased anchor number was associated with increased operative time. The average (SD) operative time for a one anchor repair was 15 (10) minutes. Each additional surgical anchor on average increased operative time by 5 min (Figure 4A).

#### 3.1.6. Effect of Surgeon Experience

Increased surgeon experience independently decreased operative time. The first 125 cases were associated with a 12-min operative time reduction (from 40 to 32 min). Over the next 200 cases, operative time increased by 3 min (from 32 to 35 min). The next 100 cases showed a decrease of 7 min (from 34 to 21 min). A 2-min reduction (from 22 min to 20 min) was evident in the remainder of the 2232 cases (Figure 3D).

#### 3.1.7. Tear Size

Smaller tear size was independently associated with reduced operative time (Figure 4B).

#### 3.1.8. Effect of Assistant Surgeon Experience

Increased assistant surgeon experience was found to independently decrease operative time. Although the effect was small, on average, the operative time reduced by 3 min (from 23 to 20 min) after assisting the main surgeon for a year.

#### 3.1.9. Sex

Female sex was found to be an independent predictor of a shorter operative time; however, its effect size was small (f^2^ = 0.007) (Table 2). Females had average (SD) operative times of 20 (12) minutes compared to 21 (13) minutes for males.

#### 3.1.10. Repair Quality

Better repair quality ranking was found to be independently associated with decreased operative time. However, the effect size of repair quality was small when all factors were accounted for (f^2^ = 0.006) (Table 2). Surgeries where repair quality was excellent on average (SD) took 18 (11) minutes compared to 35 (12) minutes for surgeries of fair repair quality (Figure 4C).

#### 3.1.11. Effect of Hospital Type

Rotator cuff repairs performed in a private day surgery were found to be faster than those performed in the public hospital. Private day surgery cases on average (SD) took 20 (12) minutes (Figure 3F). Cases performed in public hospitals on average took 25(14) minutes (Figure 3E).

#### 3.1.12. Factors That Did Not Affect Operative Time

Tissue quality, tissue mobility, tear thickness and age were found to be correlated with reduced operative time; however, they were not independent predictors when multiple regression analysis was applied. Performing a concurrent procedure was found to be correlated with increased operative time but was similarly not an independent predictor. Whether an injury was specific, the shoulder side and duration of symptoms were not found to be associated by univariate or multivariate analysis.

### 3.2. Aim 2: Sub 5-Min Arthroscopic Rotator Cuff Repair

**Figure 5:**
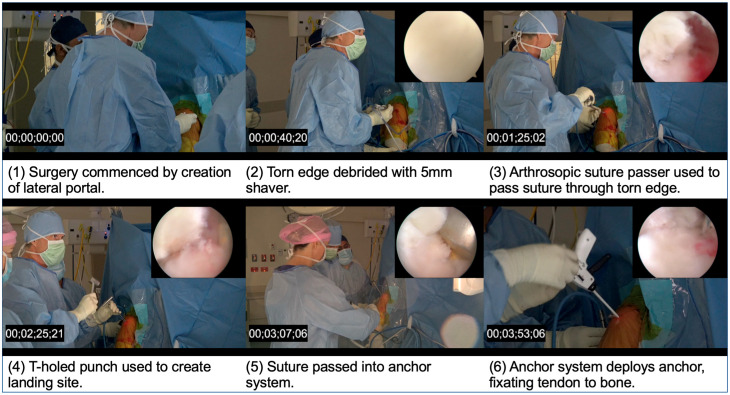
Video of 4-min arthroscopic rotator cuff repair. Adapted from “Arthroscopic undersurface rotator cuff repair in 4 min—Is it possible?” Video accessed on 16 November 2021 from: https://youtu.be/y_FvNCIz7Ds.

Video footage of consecutive arthroscopic rotator cuff repairs were recorded with the aim of capturing repair case under 5 min. Repair times for consecutive recorded cases were as follows: 5 min, 5 min, 6 min and 4 min. Footage of the 4-min repair is summarised in Figure 5.

## 4. Discussion

This study showed that the key independent factors that reduced operative time were use of undersurface repair technique, less anchors, smaller tear size, increased surgeon and assistant surgeon experience, female sex, private hospital and higher ranked repair quality. Undersurface repairs were 9 min faster on average than bursal-sided repairs. Each repair anchor added 5 min to operative time. There was an operative time reduction of 19 min after 370 cases performed by the main surgeon. Cases performed in the private day surgery centre were 5 min faster on average than cases performed in a public hospital.

Repairs where the surgery was performed with the arthroscope visualising the repair from the undersurface of the tendon were faster and less variable than repairs from the bursal side. A steeper reduction in operative time was evident when the undersurface technique was used compared to bursal-sided repairs and when both techniques were used. Bursal-sided repairs in our study on average were faster (at 25 min) than those times reported by other institutions (72–113 min) [3,4,7,12,13,14,15,16,17,18,19]. We attribute this difference to use of a knotless suture passer and anchor repair system; this system bypasses the need to tie sutures and/or create transosseous tunnels arthroscopically. The knotless technique has been shown to halve operative time compared to knotted technique [25]. We also used a single row of anchors rather than a double row or a suture bridge construct [26]. Undersurface repairs did not require bursectomy and acromioplasty, which are typically performed when the subacromial space is accessed, as in a bursal-sided repair [27]. Recently, acromioplasty has been found to be of no additional benefit in terms of patient outcome following rotator cuff repair [28,29,30]. Bypassing bursectomy may be a strength of the undersurface technique, as Morikawa et al. [31] have described the presence of pluripotent stem cells with healing potential within this space.

Reduced anchor number, reduced tear size and impaired repair quality were found to independently reduce operative time. The data also showed that cases took longer when the surgeon was less satisfied about the quality of the repair, and they were faster when the surgeon was satisfied with the quality of the repair. 

Increased surgeon experience was independently associated with decreased operative time. Two reductions in operative time were observed, corresponding to the start of the surgeon’s practice performing arthroscopic rotator cuff repairs as well as the introduction of the undersurface technique. 

Increased assistant surgeon experience was also found to be independently associated with lower operative time. There has been controversy about the role of the assistant in surgical repair of the rotator cuff. Curry et al. [4] found that the presence of surgical fellows was associated with increased operative time in a single outpatient centre treating rotator cuff tears, which they attributed to time dedicated to teaching. Conversely, a retrospective, multi-institution analysis by Green et al. [6] found that hospitals with Orthopaedic Residency Programs had lower operative times. In our study, over the course of their training, fellows on average experienced 3-min operative time reductions.

Repairs were 5 min faster on average when performed in a private day surgery setting compared to the public setting. We hypothesise that this was due to more advanced theatre equipment and relatively consistent theatre staff over the study period. Team familiarity has been shown to lower operative time in other orthopaedic procedures [32].

Clinical factors such as male sex and work-related injuries were found to be associated with increased operative time. Male sex was found to be independently predictive of increased operative time when regression was performed. However, its effect size was low, with males taking 1 min longer than females on average. As such, this may be a type 1 error caused by the high sample size of our study. A sex-based disparity in arthroscopic rotator cuff repair operative time has previously been described by Curry et al. [4] who found that male patients took 15 min longer on average than females. 

Performing concurrent procedures, tear thickness, tissue quality, tissue mobility, tear thickness, age, whether an injury was specific, shoulder side and symptom duration were not found to be associated with operative time. Previously, performing concurrent procedures with arthroscopic rotator cuff repair has been shown to prolong operative time. In particular, Curry et al. [4] found that the inclusion of distal clavicle excision, biceps tenodesis and labral debridement significantly increased operative time.

Previous studies by our institution have demonstrated good patient satisfaction and outcomes following these repairs [20,27,33].

The main limitations of this study include that it followed one surgeon, using one anchor repair system on one campus. Whilst this conferred high internal validity, this may limit external validity to other institutions, surgeons and repair systems. The study was an observational study involving a post hoc analysis of prospectively collected data. It may be worthwhile evaluating some of the factors in a prospective randomised interventional study.

A major strength was that the data was collected prospectively in a standardised systematic fashion from a large cohort of 2253 rotator cuff repair patients.

## 5. Conclusions

The key independent factors that reduced operative time were use of undersurface repair technique, use of fewer anchors in smaller tears, increased surgeon and assistant experience, and repairs performed in a private day surgery setting. Rotator cuff repairs can be performed in under 5 min through incorporation of the above measures, in particular the undersurface repair technique.

## Figures and Tables

**Table 1:** Patient demographics.

Variable	Data
Sex, *n* (%)	
Male	1259 (56%)
Female	973 (44%)
Shoulder side, *n* (%)	
Right	1338 (60%)
Left	894 (40%)
Anteroposterior tear size, mm^2^	
2–24	1778 (77%)
25–49	407 (18%)
50–74	40 (2%)
75–100	6 (0.003%)
Age, year	
Average (SD)	59 (11)
Range	15–91
Hospital type, *n* (%)	
Private	1711 (77%)
Public	242 (11%)
Missing data	279 (12%)
Repair technique, *n* (%)	
Undersurface	1260 (56%)
Bursal-side	247 (11%)
Undersurface and bursal-side	483 (22%)
Missing data	242 (11%)
Operative time, min	
Average (SD)	21 (12)
Range	3–97
Repair integrity at 6 months, *n* (%)	
Intact	1960 (88%)
Torn	272 (12%)

**Table 2:** Backwards multivariate linear regression against operative time.

Model	Unstandardised Coefficient		Standardised Coefficient Beta	t	Significance	f^2^	Effect Direction—Faster Procedures Associated with:
	B	Standard Error					
18 (Constant)		1.8		10	<0.001		
Repair technique	8.3	0.7	0.3	11	<0.001	0.08	Undersurface technique
Number of anchors	2.9	0.3	0.3	10	<0.001	0.06	Fewer anchors
Surgeon case number	−0.002	<0.001	−0.1	−4.9	<0.001	0.01	Increased surgeon case number
Tear area	0.002	0.001	0.1	4.3	<0.001	0.01	Smaller tears
Assistant case number	−0.011	0.003	−0.1	−3.9	<0.001	0.008	Increased assistant surgeon case number
Sex	1.7	0.5	0.1	3.6	<0.001	0.007	Females
Repair quality	−1.1	0.4	−0.1	−3.2	0.001	0.006	Higher ranked repair quality
Hospital type	−2.2	0.8	−0.1	−2.9	0.004	0.005	Private hospital over public hospital

Note: f^2^ is a measure of effect size in linear regression whereby f^2^ = 0.02 indicates a small effect, f^2^ = 0.15 indicates a medium effect and f^2^ = 0.35 indicates a large effect [24].

## Data Availability

The data presented in this study are available on request from the corresponding author. The data are not publicly available due to privacy reasons.

## References

[1] Jain N.B., Higgins L.D., Losina E., Collins J., Blazar P.E., Katz J.N. (2014). Epidemiology of musculoskeletal upper extremity ambulatory surgery in the United States. BMC Musculoskelet. Disord..

[2] Jain N.B., Pietrobon R., Guller U., Ahluwalia A.S., Higgins L.D. (2005). Influence of provider volume on length of stay, operating room time, and discharge status for rotator cuff repair. J. Shoulder Elb. Surg..

[3] Churchill R.S., Ghorai J.K. (2010). Total cost and operating room time comparison of rotator cuff repair techniques at low, intermediate, and high volume centers: Mini-open versus all-arthroscopic. J. Shoulder Elbow. Surg..

[4] Curry E.J., Logan C., Suslavich K., Whitlock K., Berkson E., Matzkin E. (2018). Factors impacting arthroscopic rotator cuff repair operational throughput time at an ambulatory care center. Orthop. Rev..

[5] Warrender W.J., Brown O.L., Abboud J.A. (2011). Outcomes of arthroscopic rotator cuff repairs in obese patients. J. Shoulder Elb. Surg..

[6] Green L.B., Pietrobon R., Paxton E., Higgins L.D., Fithian D. (2003). Sources of variation in readmission rates, length of stay, and operative time associated with rotator cuff surgery. J. Bone Jt. Surg..

[7] Morris J.H., Malik A.T., Hatef S., Neviaser A.S., Bishop J.Y., Cvetanovich G.L. (2020). Cost of Arthroscopic Rotator Cuff Repairs Is Primarily Driven by Procedure-Level Factors: A Single-Institution Analysis of an Ambulatory Surgery Center. Arthrosc. J. Arthrosc. Relat. Surg..

[8] Le B.T.N., Wu X.L., Lam P.H., Murrell G.A.C. (2014). Factors Predicting Rotator Cuff Retears: An Analysis of 1000 Consecutive Rotator Cuff Repairs. Am. J. Sports Med..

[9] Hill J.R., McKnight B., Pannell W.C., Heckmann N., Sivasundaram L., Mostofi A., Omid R., Hatch G.F. (2017). Risk Factors for 30-Day Readmission Following Shoulder Arthroscopy. Arthrosc. J. Arthrosc. Relat. Surg..

[10] Agarwalla A., Gowd A.K., Yao K., Bohl D.D., Amin N.H., Verma N.N., Forsythe B., Liu J.N. (2019). A 15-Minute Incremental Increase in Operative Duration Is Associated with an Additional Risk of Complications Within 30 Days After Arthroscopic Rotator Cuff Repair. Orthop. J. Sports Med..

[11] Pauzenberger L., Grieb A., Hexel M., Laky B., Anderl W., Heuberer P. (2017). Infections following arthroscopic rotator cuff repair: Incidence, risk factors, and prophylaxis. Knee Surg. Sports Traumatol. Arthrosc..

[12] Day M., Westermann R., Duchman K., Gao Y., Pugely A., Bollier M., Wolf B. (2018). Comparison of Short-term Complications After Rotator Cuff Repair: Open Versus Arthroscopic. Arthrosc. J. Arthrosc. Relat. Surg..

[13] Adla D.N., Rowsell M., Pandey R. (2010). Cost-effectiveness of open versus arthroscopic rotator cuff repair. J. Shoulder Elb. Surg..

[14] Hui Y.J., Teo A.Q.A., Sharma S., Tan B.H.M., Kumar V.P. (2017). Immediate costs of mini-open versus arthroscopic rotator cuff repair in an Asian population. J. Orthop. Surg..

[15] Baker D.K., Perez J.L., Watson S.L., McGwin G., Ponce B.A. (2017). Arthroscopic vs. open rotator cuff repair: Which has a better impact profile?. J. Shoulder Elb. Surg..

[16] Williams G., Kraeutler M.J., Zmistowski B., Fenlin J.M. (2014). No Difference in Postoperative Pain After Arthroscopic versus Open Rotator Cuff Repair. Clin. Orthop. Relat. Res..

[17] Liu J., Fan L., Zhu Y., Yu H., Xu T., Li G. (2017). Comparison of clinical outcomes in all-arthroscopic versus mini-open repair of rotator cuff tears: A randomized clinical trial. Medicine.

[18] Van der Zwaal P., Thomassen B.J.W., Nieuwenhuijse M.J., Lindenburg R., Swen J.-W.A., Van Arkel E.R.A. (2013). Clinical Outcome in All-Arthroscopic Versus Mini-Open Rotator Cuff Repair in Small to Medium-Sized Tears: A Randomized Controlled Trial in 100 Patients With 1-Year Follow-up. Arthrosc. J. Arthrosc. Relat. Surg..

[19] Ji X., Bi C., Wang F., Wang Q. (2015). Arthroscopic Versus Mini-Open Rotator Cuff Repair: An Up-to-Date Meta-analysis of Randomized Controlled Trials. Arthrosc. J. Arthrosc. Relat. Surg..

[20] Wu X.L., Baldwick C., Briggs L., Murrell G.A.C. (2009). Arthroscopic Undersurface Rotator Cuff Repair. Tech. Shoulder Elb. Surg..

[21] Duong J.K.H., Lam P.H., Murrell G.A.C. (2021). Anteroposterior tear size, age, hospital, and case number are important predictors of repair integrity: An analysis of 1962 consecutive arthroscopic single-row rotator cuff repairs. J. Shoulder Elb. Surg..

[22] Briggs L., Murrell G.A.C. (2011). Diagnostic Ultrasound: Examination of the Shoulder. Tech. Shoulder Elb. Surg..

[23] Cohen J. (1988). Statistical Power Analysis for the Behavioral Sciences.

[24] Lachenbruch P.A. (1989). Statistical Power Analysis for the Behavioral Sciences (2nd ed.). J. Am. Stat. Assoc..

[25] Burns K.A., Robbins L., LeMarr A.R., Childress A.L., Morton D.J., Wilson M.L. (2019). Rotator Cuff Repair with Knotless Technique Is Quicker and More Cost-Effective Than Knotted Technique. Arthrosc. Sports Med. Rehabil..

[26] Boyd J.A., Karas S.G., Urchek R.J., Farley K.X., Anastasio A.T., Gottschalk M.B. (2020). Factors influencing operative time in arthroscopic rotator cuff repair: A comparison of knotless single-row vs. transosseous equivalent dual-row techniques. J. Shoulder Elb. Surg..

[27] Elkins A., Lam P.H., Murrell G.A.C. (2019). A Novel, Fast, Safe, and Effective All-Inside Arthroscopic Rotator Cuff Repair Technique: Results of 1000 Consecutive Cases. Orthop. J. Sports Med..

[28] Chahal J., Mall N., MacDonald P.B., Van Thiel G., Cole B.J., Romeo A.A., Verma N.N. (2012). The Role of Subacromial Decompression in Patients Undergoing Arthroscopic Repair of Full-Thickness Tears of the Rotator Cuff: A Systematic Review and Meta-analysis. Arthrosc. J. Arthrosc. Relat. Surg..

[29] Singh C., Lam P.H., Murrell G.A.C. (2021). Effect of Acromioplasty on Postoperative Pain Following Rotator Cuff Repair. HSS J..

[30] Nam J.-H., Park S., Lee H.-R., Kim S.H. (2018). Outcomes After Limited or Extensive Bursectomy During Rotator Cuff Repair: Randomized Controlled Trial. Arthrosc. J. Arthrosc. Relat. Surg..

[31] Morikawa D., Johnson J.D., Kia C., McCarthy M.B.R., Macken C., Bellas N., Baldino J.B., Cote M.P., Mazzocca A.D. (2019). Examining the Potency of Subacromial Bursal Cells as a Potential Augmentation for Rotator Cuff Healing: An In Vitro Study. Arthrosc. J. Arthrosc. Relat. Surg..

[32] Maruthappu M., Duclos A., Zhou C.D., Lipsitz S.R., Wright J., Orgill D., Carty M.J. (2016). The impact of team familiarity and surgical experience on operative efficiency: A retrospective analysis. J. R. Soc. Med..

[33] Rubenis I., Lam P.H., Murrell G.A. (2015). Arthroscopic Rotator Cuff Repair Using the Undersurface Technique: A 2-Year Comparative Study in 257 Patients. Orthop. J. Sports Med..

